# Late onset of two concurrent and dissociated arrhythmias in a transplanted heart

**DOI:** 10.1016/j.ipej.2024.09.009

**Published:** 2024-09-25

**Authors:** Gala Caixal, Paz Garre, Lluis Mont, Ivo Roca-Luque

**Affiliations:** a Unitat d’Arrítmies, Institut Clínic Cardiovascular (ICCV), Hospital Clínic, Universitat de Barcelona, Barcelona, Catalonia, Spain; b Institut d’Investigacions Biomèdiques August Pi i Sunyer (IDIBABS), Barcelona, Catalonia, Spain; c Centro de Investigación Biomédica en Red de Enfermedades Cardiovasculares (CIBERCV), Instituto de Salud Carlos III, Madrid, Spain

**Keywords:** High-resolution mapping, Heart transplantation, Atrial tachycardia, Catheter ablation, Right atrium, Bicaval technique

## Abstract

A 53-year-old patient with a history of heart transplant is referred for atrial tachycardia ablation. Two dissociated concomitant rhythms are observed: a focal atrial tachycardia in the donor atrium and atrial fibrillation in the remaining recipient atrium.

## 1 Introduction

Atrial arrhythmias after orthotopic heart transplantation (OHT) are common and well documented [1]. The most common are the macro-reentrant type, since the isolation of the pulmonary veins and autonomic denervation lead to a low rate of atrial fibrillation (AF) and focal atrial tachycardia (AT) in these patients [2]. Some cases of AT have been described in transplant patients, however in most of them the tachycardia originated in the recipient atrium and was conducted to the donor's through the suture line [3,4].

We present a case of OHT performed with a bicaval technique that presented 2 different concurrent arrhythmias in late follow-up; a focal AT originating in the donor's right atrium (RA) and AF located in the remaining atrial area of the recipient.

## 2 Case report

A 53-year-old patient, undergoing bicaval OHT in 2009 for arrhythmogenic biventricular dysplasia, was referred for catheter ablation of a medically uncontrolled incessant atrial arrhythmia. During the years after transplantation, the patient had not experienced rejection but developed severe vascular disease of the graft requiring prior percutaneous and surgical revascularization and was currently awaiting treatment for an asymptomatic progressive lesion in the left main coronary artery.

**Fig. 1:**
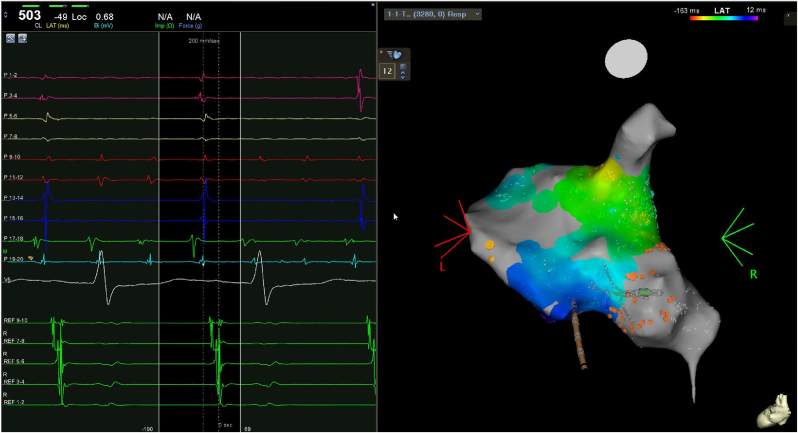
Right atrial activation mapping (posteroanterior view) using the CARTO contact mapping system and intracardiac electrograms on the PentaRay catheter (P) and on the decapolar catheter within the coronary sinus (CS) (REF). The remaining recipient atrium (orange dots) presented atrial fibrillation (P 9–12 and P 17–20) and the donor atrium was in focal atrial tachycardia (REF, P 1–8 and P 13–16).

**Fig. 2:**
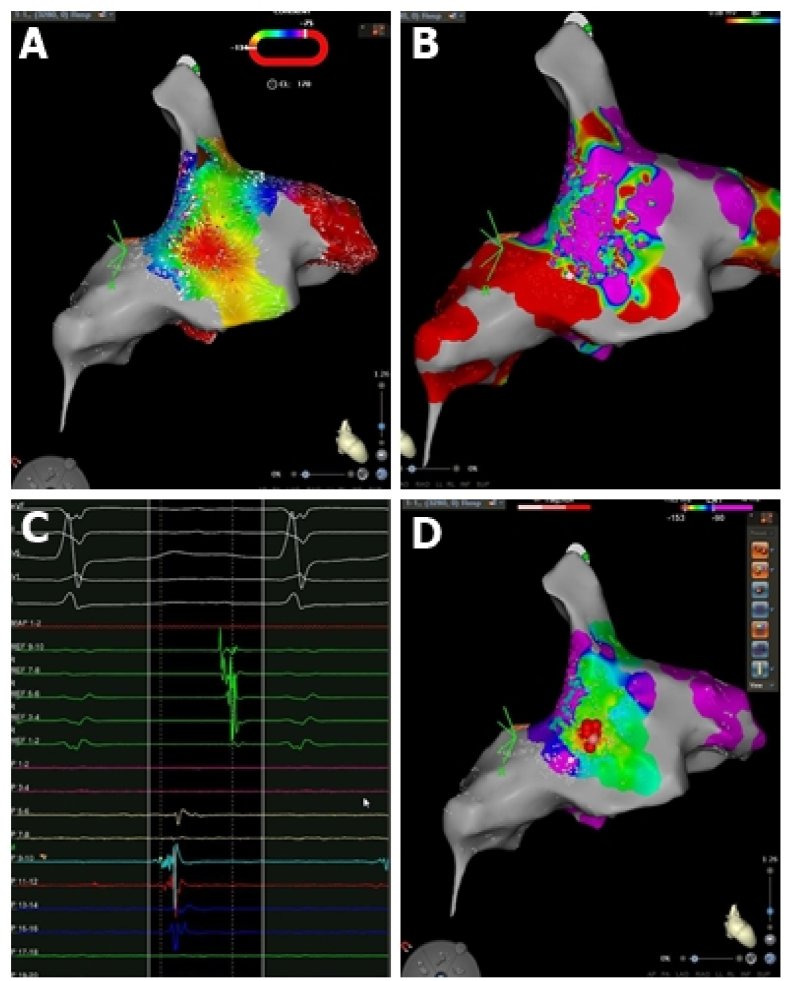
Atrial tachycardia in the donor's right atrium. A) Activation map and coherent. B) Voltage map. C) Maximum precocity in the intracavitary electrograms (P 9–10) D) Final ablation set (red dots) at the maximum precocity site.

**Fig. 3:**
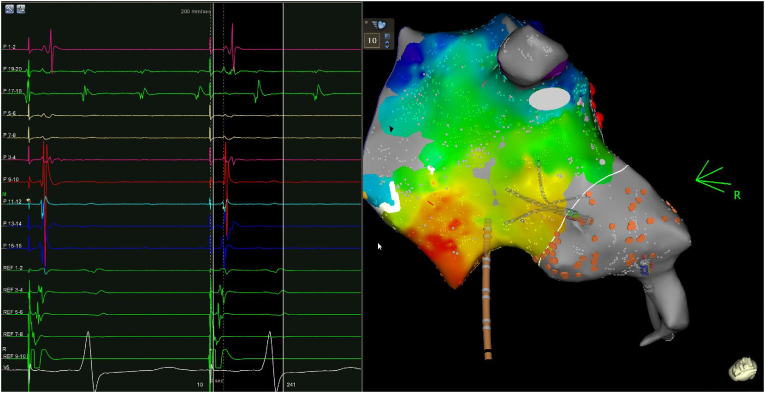
Mapping of the right atrium after atrial tachycardia ablation. The native atrium (orange dots) persisted in atrial fibrillation without any change (P 17–20). The donor atrium was in sinus rhythm and this figure shows its activation under coronary sinus pacing (REF 9–10), confirming the electrical dissociation of both atrial parts.

One 6F 10-pole catheter was inserted through the femoral vein and advanced into the coronary sinus (CS). Electroanatomical mapping of the RA was performed with CARTO 3 (Bioscense Webster, USA) and a PentaRay multipolar catheter (Bioscense Webster, USA). Intracardiac recordings revealed a large atrial area of low voltage (less than 0.1mV), probably the surgical scar, at the junction between the RA and the inferior vena cava (IVC), as well as the presence of two different and dissociated arrythmias; The recipient's remaining atrium was in AF and the donor's atrium presented an AT with a cycle length (CL) of 440 ms (Fig. 1). This AT was the clinical arrythmia and could not be entrained or interrupted after several attempts with atrial pacing at a CL of 400–430 ms. The activation map showed centrifugal activation suggesting a focal AT located in the lower anterolateral area of the RA, near the low-voltage area (Fig. 2A and B). In this area, radiofrequency was administered with an open-irrigation magnetic ablation catheter (Navistar, Thermocool, Bioscense Webster, USA) with instant termination of the tachycardia (Fig. 2C and D), being subsequently not inducible basally or under isoprenaline perfusion. Finally, a remap of the RA was performed, pacing from the distal CS, observing the persistence of AF in the recipient's atrium and confirming the electrical disconnection of the 2 rhythms (Fig. 3). Since the rhythm of the recipient's atrium was not clinically relevant to the management of the patient, it was decided not to perform treatment on the AF observed in this area.

## 3 Discussion

Atrial arrhythmias in the donor atrium late after heart transplantation are most commonly macro-reentrant and often rely on the cavotricuspid isthmus or incorporate the surgical incision as a boundary. However, sometimes focal AT can originate in low-voltage areas or border adjacent to the atrio-atrial anastomosis after biatrial technique [5,6], as well as be the result of conduction from the recipient's atrium through the suture line [3]. In our case, the incessant form of the tachycardia, the difficulty to entrain or terminating it with pacing and the centrifugal activation suggested a focal tachycardia probably due to abnormal automatism, although microreentry with entry block cannot be ruled out. This focal mechanism here has several possible explanations.

The OHT with bicaval anastomosis attempts to avoid incisions in the RA and reduce arrhythmogenic substrate compared to standard biatrial anastomosis [7,8]. In this patient, the available surgical report explains having used the bicaval technique but without providing the exact boundaries of junction between donor and recipient. After visualizing the large area of low-voltage on the RA mapping, it was concluded that some of the auricular tissue could be covered by the suture line. This could have increased the possibility of reentry surrounding the scar, but also of focal automaticity in the adjacent area.

On the other hand, some focal tachycardias in the donor atrium in late follow-up have been related to transplant rejection or vasculopathy [2,9]. Secondary substrate abnormalities can cause direct electrophysiological alteration of the atrial myocardium, atrial stretch and interstitial fibrosis, allowing and promoting wavelet reentry and automatic responses. In our patient, endomyocardial biopsy showed no rejection, however, his background of cardiac allograft vasculopathy (CAV) could have contributed to the development of AT in the donor atrium. The pathogenesis of CAV is not yet fully understood, but it seems to be the result of a complex interaction between immunological and non-immunological factors that induce endothelial damage and represents the main late cause of morbidity and mortality, affecting almost half of patients at ten years after transplantation. In such cases, it is important to distinguish CAV from rejection reaction (cellular or humoral) and non-specific graft dysfunction. Rejection reactions are diagnosed on endomyocardial biopsy; however, this technique has limited sensitivity for recognizing vasculopathy because it only samples the smallest intramyocardial arteries and arterioles, which often do not show histological features of CAV. Furthermore, rejection may be patchy and the biopsy site may not reflect ongoing rejection in other areas of the myocardium. For all these reasons, a rejection component cannot be ruled out in our patient despite the negative result of the endomyocardial biopsy.

After heart transplantation, the presence of two different tachycardias isolated from each other due to conduction line block is a rare and challenging situation in electrophysiology. Especially when a surgical report where the incisions are clearly detailed is not available, the use of 3D navigators and high-density mapping is an essential tool. Although we will never know with certainty why our patient presented with this focal atrial tachycardia, rapid and high-resolution mapping allowed clear delineation of the atrial substrate and the diagnosis of two simultaneous tachycardias. This allowed precise and effective ablation of the clinical arrhythmia, leaving untreated AF that had no clinical impact and the ablation of which would have unnecessarily lengthened the procedure.

## 4 Conclusions

We present the case of a patient with two concurrent late-onset atrial arrhythmias after heart transplantation: a focal AT in the donor RA and AF in the recipient, both being electrically disconnected from each other. To our knowledge, this is the first report of these two arrhythmias presented with a high-density electroanatomical map after bicaval heart transplantation.

## Funding

This research did not receive any specific grant from funding agencies in the public, commercial, or not-for-profit sectors.

## Conflict of interest

The authors have no conflicts to disclose.

## Ethical conflicts

There are no ethical conflicts.

## Declaration of competing interest

The authors declare that they have no known competing financial interests or personal relationships that could have appeared to influence the work reported in this paper.
